# Evaluation of body-surface-area adjusted dosing of high-dose methotrexate by population pharmacokinetics in a large cohort of cancer patients

**DOI:** 10.1186/s12885-021-08443-x

**Published:** 2021-06-20

**Authors:** Usman Arshad, Max Taubert, Tamina Seeger-Nukpezah, Sami Ullah, Kirsten C. Spindeldreier, Ulrich Jaehde, Michael Hallek, Uwe Fuhr, Jörg Janne Vehreschild, Carolin Jakob

**Affiliations:** 1 Department I of Pharmacology, Faculty of Medicine and University Hospital Cologne, Center for Pharmacology, University of Cologne, Gleueler Str 24, 50931 Cologne, Germany; 2 Institute of Pharmacy, Clinical Pharmacy, University of Bonn, Bonn, Germany; 3 Department I of Internal Medicine, Faculty of Medicine and University Hospital Cologne, University of Cologne, Cologne, Germany; 4 Hospital Pharmacy, University Hospital Cologne, Cologne, Germany; 5 German Center for Infection Research (DZIF), Partner site Bonn-Cologne, Cologne, Germany; 6 Department of Internal Medicine, Hematology and Oncology, Faculty of Medicine and University Hospital of Frankfurt, Goethe University Frankfurt, Frankfurt am Main, Germany

**Keywords:** Methotrexate, Pharmacokinetics, Covariates, Dosing

## Abstract

**Background:**

The aim of this study was to identify sources of variability including patient gender and body surface area (BSA) in pharmacokinetic (PK) exposure for high-dose methotrexate (MTX) continuous infusion in a large cohort of patients with hematological and solid malignancies.

**Methods:**

We conducted a retrospective PK analysis of MTX plasma concentration data from hematological/oncological patients treated at the University Hospital of Cologne between 2005 and 2018. Nonlinear mixed effects modeling was performed. Covariate data on patient demographics and clinical chemistry parameters was incorporated to assess relationships with PK parameters. Simulations were conducted to compare exposure and probability of target attainment (PTA) under BSA adjusted, flat and stratified dosing regimens.

**Results:**

Plasma concentration over time data (2182 measurements) from therapeutic drug monitoring from 229 patients was available. PK of MTX were best described by a three-compartment model. Values for clearance (CL) of 4.33 [2.95–5.92] L h^− 1^ and central volume of distribution of 4.29 [1.81–7.33] L were estimated. An inter-occasion variability of 23.1% (coefficient of variation) and an inter-individual variability of 29.7% were associated to CL, which was 16 [7–25] % lower in women. Serum creatinine, patient age, sex and BSA were significantly related to CL of MTX. Simulations suggested that differences in PTA between flat and BSA-based dosing were marginal, with stratified dosing performing best overall.

**Conclusion:**

A dosing scheme with doses stratified across BSA quartiles is suggested to optimize target exposure attainment. Influence of patient sex on CL of MTX is present but small in magnitude.

**Supplementary Information:**

The online version contains supplementary material available at 10.1186/s12885-021-08443-x.

## Introduction

Methotrexate (MTX) is considered an efficacious, cost-effective and acceptably safe drug for the treatment of many hematological/oncological disorders and autoimmune diseases [1]. The folate analogue MTX acts as an antineoplastic agent via competitive inhibition of dihydrofolate dehydrogenase, resulting in depletion of purines and thymidylate leading to impairment of DNA synthesis [2, 3]. The drug can be administered via multiple routes of administrations and has a wide variation in dosing regimens including low (< 50 mg/m^2^), intermediate (50–500 mg/m^2^) and high (> 500 mg/m^2^) dose regimens [1, 4]. The pronounced inter-individual variability (IIV) of PK and toxicity of MTX [5–7] renders individualization of dosing regimens difficult.

Hepatic metabolism accounts for a considerably lower fraction of its clearance (CL) compared to renal elimination, as the main fraction (80–90%) of the drug is primarily eliminated via glomerular filtration and active tubular secretion [8, 9]. Nephrotoxicity associated with MTX impairs its CL, leading to further aggravation of toxicity such as myelosuppression and mucositis. In subjects with extracellular fluid accumulations, the drug has been shown to undergo delayed elimination [10]. A recent in vitro study by Euteneuer et al. [11] showed a sex-dependent regulation of renal transport proteins, which might play a role in the CL of MTX. To handle the variability associated with MTX exposure, monitoring of its plasma concentrations (therapeutic drug monitoring, TDM) and serum creatinine (SCr) is recommended to safeguard a relatively constant drug exposure with an acceptable risk/benefit ratio particularly in patients with impaired renal function [12]. Furthermore, MTX dosing is often guided by body surface area (BSA) estimates to account for body size-related differences in CL and volume of distribution (V). However, concerns regarding potential under- and over-exposure in certain patient groups, such as with obesity, have been expressed [13]. BSA is furthermore a highly variable measure that depends on the arbitrary choice of a BSA equation [14]. Thus, further clarification of the clinical implications of BSA based dosing for MTX is required.

Modeling of PK data has the potential to optimize TDM, where tailored dose adjustments can be made according to model-predicted concentrations of a drug [15]. Bayesian population PK analysis has been used to assist TDM guided dose adjustments for MTX [15]. In addition, population PK analysis provides the possibility to identify and quantify covariate effects on drug exposure [16, 17]. This may provide a better understanding of drug’s pharmacology and assist adjustments in dosage regimen according to patient’s individual characteristics e.g., renal/hepatic function, genotype of drug metabolizing enzymes or transporters, and/or anthropometric characteristics. Models capturing covariate relationships have been found useful in oncology for individualized dose adaptations such as in case of busulfan, topotecan and docetaxel [16].

The current study was aimed to identify and evaluate covariates influencing PK of MTX, particularly patient sex and body surface area (BSA), by developing a population PK model using the TDM data collected from patients with hematological and solid malignancies. The model was further aimed to be used for the evaluation of the ongoing clinical practice of administering MTX based on individual BSA via a simulation study.

## Methods

### Patients, treatment and sampling

MTX plasma concentration and covariate data was obtained from the Cologne Cohort of Neutropenic Patients (CoCoNut) [18]. Experimental protocols were approved by the local ethics committee (name and email address: Ethics Committee of the Faculty of Medicine, University of Cologne, Cologne, Germany, ek-med@uni-koeln.de; date of approval: 14.01.2014, approval file number: 13–108). All methods were performed in accordance with the local and international guidelines and regulations. Data from neutropenic patients (neutrophils < 500 /mm^3^) with hematological malignancies or solid tumors and treated with high-dose MTX at the Department I of Internal Medicine, University Hospital of Cologne, between January 2005 and February 2018 were considered. The data from clinical laboratory was imported via Health Level Seven from the laboratory information system. The dosing information was imported from the integrated software for chemotherapy using a csv export. Further patient characteristics were documented manually in the CoCoNut database.

MTX was administered via 4 h or 24 h intravenous infusions depending on underlying malignancy. TDM was routinely performed at 42 h and 48 h post-dose for both the 4 h and 24 h protocols, while an additional sample was scheduled for 4 h MTX infusion at 24 h. If target plasma concentration exceeded the desired thresholds (> 1 μmol/L at 42 h and > 0.3 μmol/L at 48 h), TDM was performed at least every 6 h. These thresholds reflect the internal guidance document developed to translate the available heterogenous evidence [10, 19] to an actionable recommendation also appropriate for the organisational conditions in our hospital. On the same basis, for the 24 h MTX infusion, leucovorin rescue was routinely performed with 30 mg/m^2^ (after 42 h and 48 h) and 15 mg/m^2^ (after 54 h and 60 h). If the desired plasma concentration of MTX was not reached, leucovorin was administered every 6 h at a dose (mg) equivalent to the product of MTX plasma concentration (μmol/L) and body patient weight (kg).

MTX plasma concentrations were quantified using competitive immunoassays with 0.009 μmol/L as the lower limit of quantification (LLOQ). Demographic covariates included patient’s age, sex, weight and height. Covariate data from clinical chemistry analysis included SCr, plasma total bilirubin (BT), γ-glutamyltransferase (GGT), uric acid concentrations, absolute leukocyte counts (WBC), and BSA.

Dosing, concentration and covariate data was subjected to screening prior to PK analysis. R (version 3.5.1) was used to prepare the dataset for model development. Dataset preparation was assisted by visual inspection of individual concentration time profiles. Patients with missing dosing information at treatment initiation were identified for exclusion from subsequent analysis. Subjects with missing dosing information during the treatment were flagged and concentration measurements at time points subsequent to the missing dosing information were excluded. Due to the significant amount of missing covariate data throughout the treatment course, the covariate evaluation was based on baseline covariate data for the start of treatment.

### PK model development

Data were analyzed by the nonlinear mixed effects modeling approach using NONMEM 7.4.3 (ICON, Development Solutions, Elliot City, MD, USA). Perl speaks NONMEM (PsN), Pirana and Xpose4 were used to assist model development, evaluation and post processing [20–22]. Structural model development. A combination of iterative two-stage (ITS) and first order conditional estimation with interaction (FOCE-I) methods was applied for parameter estimation. Likelihood ratio tests (LRT) or the Akaike information criterion (AIC) were used for the evaluation of nested and non-nested models, respectively. A nested model with fewer parameters or a decrease in objective function value (OFV) by 3.84 (i.e., *p* < 0.05, one degree of freedom) was given preference. The model with a lower AIC value in case of non-nested models was preferred.

Model evaluation criteria comprised of plausibility of parameter estimates, reduction in unexplained and residual variability, shrinkage and precision in parameter estimates. Visual inspection through goodness of fit (GOF) plots included observed versus individual/population predicted concentrations (IPRED/PRED) over time. Residual error models were evaluated with the help of conditional weighted residuals (CWRES) versus observed concentrations and versus time after first dose (TAFD). Numerical predictive checks (NPCs) were used for further assessment by comparing the empirical cumulative distribution function of the observed concentrations with the theoretical cumulative distribution, computed from simulated data.

Compartmental analysis was performed in a stepwise manner. IIV was incorporated using exponential terms (η_iiv_) which describes the deviation of PK parameter values of an individual from the population estimate [17]. Interoccasion variability (IOV), defined as the variability between individual cycles of MTX therapy, was incorporated in the model via random effects (η_iov_) [23]. The PK parameter P in a specific subject was parametrized as shown in Eq. 1.

1 $$ \mathrm{P}=\uptheta \times {\mathrm{e}}^{\upeta_{\mathrm{iiv}}+{\upeta}_{\mathrm{iov}}} $$

Where θ is a fixed effect, representing the median PK parameter in the population. Additive, proportional and combined error models were tested to estimate the residual unexplained variability (RUV).

### Covariate model development

Covariate data was analyzed to identify covariate-parameter relationships. Covariate preselection was performed considering scientific plausibility as an essential criterion. Graphical evaluation of covariates was performed including CWRES vs covariate, empirical Bayes estimates (EBEs) versus covariate, and covariate versus covariate plots. Significance of covariate relationship was principally guided by decrement in OFV and/or unexplained variability. A stepwise covariate evaluation was carried out as follows. At each step, the covariate providing the largest reduction in OFV was included (forward inclusion) or the covariate providing the lowest increase in OFV was eliminated (backward elimination). Selection criteria were a ∆OFV of 3.84 (*p* < 0.05) for forward inclusion and a ∆OFV of 6.63 (*p* < 0.01) for backward elimination.

Continuous covariates were included as linear relationships (Eq. 2) or power relationships (Eq. 3) centered around their median values. BSA effect was centered around the typical value of 1.73 m^2^.

2 $$ {\mathrm{Covariate}}_{\mathrm{effect}}=1+\left({\mathrm{Covariate}}_{\mathrm{i}}-{\mathrm{Covariate}}_{\mathrm{median}}\right)\times {\uptheta}_{\mathrm{Covariate}} $$

3 $$ {\mathrm{Covariate}}_{\mathrm{effect}}={\left(\raisebox{1ex}{${\mathrm{Covariate}}_{\mathrm{i}}$}\!\left/ \!\raisebox{-1ex}{${\mathrm{Covariate}}_{\mathrm{median}}$}\right.\right)}^{\uptheta_{\mathrm{Covariate}}} $$

Categorical relationships were given as Covariate_effect_ = 1 + Covariate_i_ × θ_Covariate_, where Covariate_i_ is the individual covariate value in the i^th^ subject and θ_Covariate_ represents the effect size of the covariate relationship to a PK parameter. Covariate inclusion and evaluation criteria are presented in the supplementary material.

### Evaluation of BSA-based, flat and stratified dosing regimens

Stochastic simulations were designed using the final model, including covariates, for the comparative evaluation of drug exposure under BSA-based (linear scaling using BSA), flat and stratified dosing 24 h infusion regimens. Stratified dosing regimens comprised of 3 BSA-based stratifications i.e., subjects at lower and upper BSA extremes (< 25th and > 75th BSA percentiles) as well as the middle (25th–75th percentile) proportion of population. No further differences in virtual patient characteristics were part of the simulated populations. The three sets of simulated populations differed only in the administered dosing regimens. Target for an adequate dosing regimen included two criteria. First, plasma concentrations should not exceed 1.0 and 0.3 μmol/L at 42 h and 48 h after the start of infusion, respectively, based on the current TDM protocol at the University Hospital, Cologne. Second, the achieved AUC should be in the range of ±30% of the AUC of a subject with a typical BSA of 1.73m^2^.

## Results

### Patient and treatment characteristics

In total, 229 cancer patients (83 females) with 2182 plasma concentration measurements were included in the PK analysis. The majority of patients received 4 h and 24 h infusions, while 18 patients occasionally received 12 h and 48 h infusions. Only a single patient received a 72 h infusion. A median of 3 dosing cycles (range, 1–9) per patient were part of the available data. The number of plasma concentration measurements per patient ranged from 1 to 65 with a median of 7 measurements. Patient and clinical laboratory parameters are summarized in Tables 1 and 2.

**Table 1:** Population characteristics. Median and range for measured values are shown

Characteristics	Total ***n*** = 229	Females ***n*** = 83	Males ***n*** = 146
Age (years)	58 [19, 82]	66 [19, 77]	51 [19, 82]
Weight (kg)	78.4 [41.5, 227	70.2 [41.5, 96.5]	84.3 [50.0, 227]
Height (cm)	176 [154, 203]	167 [154, 180]	180 [162, 103]
Body surface area (m^2^)	1.96 [1.34, 3.42]	1.80 [1.34, 2.11]	2.06 [1.54, 3.42]
Body mass index (kg/m^2^)	25.4 [15.7, 66.3]	25.3 [15.7, 38.7]	26.0 [17.3, 66.3]
Serum creatinine (mg/dL)	0.74 [0.36, 1.66]	0.67 [0.38, 1.34]	0.84 [0.36, 1.66]
Total plasma bilirubin (mg/dL)	0.48 [0.09, 2.90]	0.45 [0.09, 1.60]	0.50 [0.09, 2.90]
Plasma γ-glutamyltransferase (mg/dL)	69.8 [14.0, 442]	67.2 [16.0, 442]	72.1 [14, 441]
Plasma urea (mg/dL)	32.0 [2.90, 949]	29.1 [2.90, 58.0]	47.0 [12.0, 949]
Absolute leucocyte count (×10^9^/L)	6.28 [0.05, 61.1]	5.77 [0.29, 33.29]	6.63 [0.05, 61.1]

Body surface area was computed using Du Bois Formula [46]

Normal levels Serum creatinine: females 0.5–0.9 mg/dL; males 0.5–1.1 mg/dL; total plasma bilirubin: 0–1.2 mg/dL; plasma γ-glutamyltransferase: females 0–40 mg/dL; males 0–60 mg/dL; plasma urea: 0–50 mg/dL; absolute leucocyte count: 4.4–11.3 × 10^9^/L; Missing data was interpolated (last observation carried forward/backward)

**Table 2:** Population disease characteristics

Tumor type	n
Solid tumors	
Sarcoma	4
Carcinoma	2
Hodgkin lymphoma	5
Non-Hodgkin lymphoma	9
Leukemia / very aggressive Non-Hodgkin lymphoma	
Acute lymphoblastic leukemia	64
Acute myeloid leukemia	1
Others	43
Low aggressive Non-Hodgkin lymphoma	101

Diagnoses were defined as following ICD-10 codes

Sarcoma: C30.1, C34.1, C34.3, C34.8, C40.0, C40.2, C41.2, C41.3, C41.9

Carcinoma: C49.9, C58

Hodgkin lymphoma: C81.1, C81.2, C81.7, C81.9

Non-Hodgkin lymphoma: C71.0, C71.1, C71.2, C71.8, C78.2, C79.3, C79.5

Acute lymphoblastic leukemia: C91.00, C91.01, C91.50, C91.51, C91.80, C91.81

Acute myeloid leukemia: C92.00, C92.01, C94.70, C95.00

Others: C83.1, C83.7, C83.8, C84.4, C84.5, C86.2, C91.40, C91.60, C91.61

Low aggressive Non-Hodgkin lymphoma: C82.2, C82.4, C82.7, C82.9, C83.0, C83.3, C83.5, C85.1, C85.2, C85.7, C85.9, C91.10

### PK model

A three-compartment model with linear elimination adequately described MTX plasma concentrations (Supplementary Figure 1 & 2). We decided to use a linear CL model instead of a model with an additive nonlinear CL component (combined model) for the subsequent evaluations, although the latter provided a better fit with (∆OFV of − 70 points). The fraction of CL contributed by the linear component in the combined model was 4.77 L/h, whereas nonlinear CL solely contributed 0.42 L/h at median MTX concentrations (2.20 μmol/L). Furthermore, run times were distinctly longer (~ 60 h compared to ~ 1 h), preventing from a proper covariate analysis, and parameter estimation was unstable. Estimates from the combined model with linear and nonlinear CL components are presented in the Supplementary Table.

RUV was appropriately described by a combined (additive and exponential) error model. Mean PK parameters with 95% CI and RSE obtained from the bootstrap analysis (1000 samples) are presented in Table 3.

**Table 3:** Population PK parameter estimates from bootstrap analysis

	Median	% RSE	95% CI
**PK parameters**			
CL (L h^− 1^)	4.33	21.4	2.95–5.92
V_1_ (L)	4.29	52.5	1.81–7.33
V_2_ (L)	2.51	61.1	0.82–5.37
V_3_ (L)	2.36	35.5	0.65–7.25
Q_1_ (L h^− 1^)	0.37	38.2	0.16–0.62
Q_2_ (L/h)	0.02	51.38	0.01–0.06
**Covariate effects on CL**			
SCr (mg^− 1^ dL)	− 0.49	−21.1	− 0.31 - -0.08
Age (year^−1^)	− 0.18	− 37.7	− 0.30 - 0.05
Sex (fractional decrease in females)	−0.16	−35.4	− 0.25 - -0.07
BSA (m^− 2^)	0.23	151	−0.33 - 0.67
**IIV (ω**^**2**^**)**			
CL	0.11	17.1	0.08–0.14
V_1_	1.34	144	0.83–2.27
COV (CL, V_1_)	0.29	77.6	0.18–0.39
**IOV (ω**^**2**^**)**			
CL	0.09	15.7	0.07–0.11
V_1_	–	–	–
**RUV (σ**^**2**^**)**			
Additive error	0.02	17.3	0.02–0.03
Exponential error	0.26	6.93	0.24–0.29

*PK* pharmacokinetic, *RSE* relative standard error, *CI* confidence interval, *CL* clearance, *V*_*1*_ central volume of distribution, *V*_*2*_ and *V*_*3*_ peripheral volumes of distribution, *Q*_*1*_ and *Q*_*2*_ inter-compartmental clearances, *AUC* Area under the curve, *SCr* Serum, Creatinine, *IIV* inter-individual variability, *COV* covariance, *IOV* inter-occasion variability, *RUV* residual unexplained variability

### Covariate analysis

SCr was found to be a significant covariate on CL with an OFV reduction by 191. Inclusion of patient’s sex and age on CL further improved the model fit (∆OFVs of 32.0 and 13.0, respectively). Inclusion of BSA provided a significant reduction in OFV by 4.40 on CL. A 16% [7–25%] lower CL was estimated in females. Reduction in IIV of individual parameters was limited, with a decrease in 2.40, 0.56 and 1.44 (%) after inclusion of SCr, age and sex, respectively. IIV and IOV on CL in the covariate model were 29.7 and 23.1%, respectively. The resulting equation for the individual CL (CL_i_) is shown in Eq. 4.

4 $$ {\mathrm{CL}}_{\mathrm{i}}=4.52{\left(\raisebox{1ex}{${\mathrm{SCr}}_{\mathrm{i}}$}\!\left/ \!\raisebox{-1ex}{$0.74$}\right.\right)}^{-0.49}{\left(\raisebox{1ex}{${\mathrm{Age}}_{\mathrm{i}}$}\!\left/ \!\raisebox{-1ex}{$58$}\right.\right)}^{-0.18}{\left(\raisebox{1ex}{${\mathrm{BSA}}_{\mathrm{i}}$}\!\left/ \!\raisebox{-1ex}{$1.73$}\right.\right)}^{-0.23}\left(1+{\mathrm{Sex}}_{\mathrm{i}}\times -0.16\right) $$

Where, sex was coded as 0 for males and 1 for females. Estimates for covariate relationships are summarized in Table 3.

### BSA-based versus flat and stratified dosing regimens

**Fig. 1:**
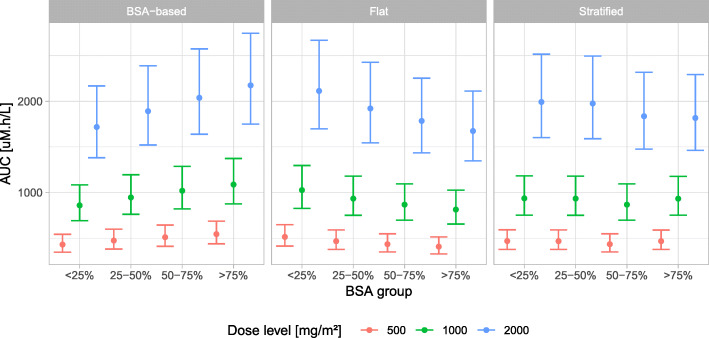
Distribution of simulated area under the curve (AUC; median with 95% CI) across body surface area (BSA) quartiles for BSA-based, flat and stratified dosing. Description of doses under each regimen is presented in Table 4

**Fig. 2:**
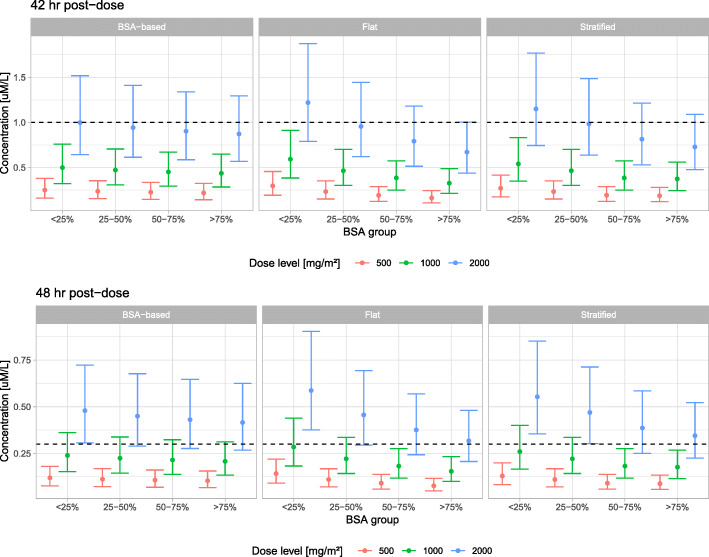
Distribution of simulated plasma concentrations (median with 95% CI) across body surface area (BSA) quartiles for BSA-based, flat and stratified dosing. Dashed horizontal lines represent the desired threshold plasma concentrations (< 1 μmol/L at 42 h post-dose and < 0.3 μmol/L at 48 h post-dose). Description of doses under each regimen is presented in Table 4

Figures 1 and 2 presents the distribution of AUC and plasma concentrations respectively, in the virtual population stratified by BSA quartiles for BSA-based, flat and stratified dosing regimens. A gradual increase in MTX AUC with increase in BSA was associated with BSA-based regimen, while the contrary was observed with flat dosing regimen. Stratified dosing displayed a consistent AUC across all the BSA quartiles. Concerning the decline of MTX concentrations until 42 and 48 h postdose, the higher clearance for higher BSA values more than compensated for the concentration differences between BSA-based and stratified dosing just at the end of the infusions.

**Fig. 3:**
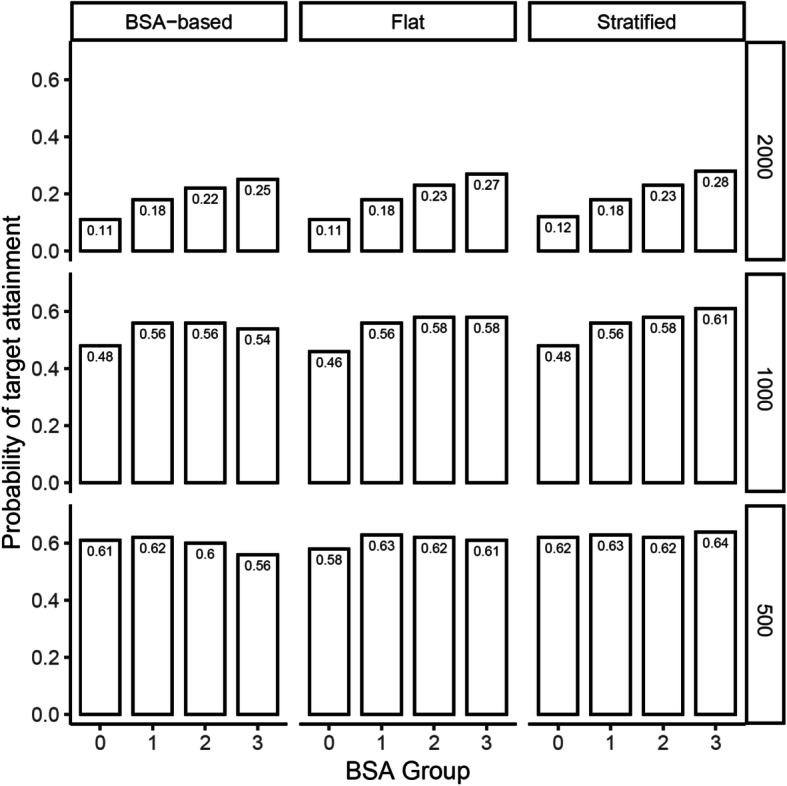
Probability of target attainment (PTA) across body surface area (BSA) groups under BSA-based, flat and stratified dosing regimens. Reference BSA-based dose levels range from 500 to 2000 mg/m^2^. Numbers in the bars represent respective PTA values. (0 = < 25%, 1 = 25–50%, 2 = 50–75%, 3 = > 75%). Description of doses under each regimen is presented in Table 4

The percentage of subjects attaining both the target criteria (probability of target attainment; PTA) was calculated for dose levels of 500, 1000 and 2000 mg/m^2^ (reference dose). An optimized flat dosing regimen, i.e. a regimen in which each subject received the same dose, was identified by simulating a range of doses and choosing the dose that provided the highest PTA. Subsequently, the procedure was repeated with doses stratified according to the BSA groups (lower extreme: < 25%, middle proportion: 25–75% and upper extreme > 75%) and the above-mentioned dose optimization was repeated for each of the three BSA regions separately. Thus, the stratified dosing approach resulted in three separate doses, corresponding to the three defined BSA groups. Figure 3 presents the PTA across the BSA groups for respective dose levels under BSA-based, flat and stratified dosing regimens. Stratified dosing provided marginally higher PTA for both the upper and lower BSA extremes compared to BSA-based and flat dosing, respectively. Based on simulation results, selection of doses with the highest PTA (comparable to BSA-based doing regimen) identified under flat and stratified dosing regimens are presented in Table 4.

**Table 4:** Selection of stratified doses with highest probability of target attainment (PTA) compared to that of the body surface area (BSA)-based and flat doses of MTX administered as 24 h continuous infusion

BSA-based	Flat	Stratified		
		< 25% (< 1.7m^**2**^)	25–75% (1.7–2.12 m^**2**^)	> 75% (> 2.12 m^**2**^)
500 mg/m^2^	850 mg	775 mg	850 mg	975 mg
1000 mg/m^2^	1700 mg	1550 mg	1700 mg	1900 mg
2000 mg/m^2^	3400 mg	3000 mg	3400 mg	3800 mg

## Discussion

High-dose MTX is essential in cancer therapies despite its high toxicity. However, the management of delayed MTX elimination challenges clinicians to prevent potentially life-threatening MTX-associated toxicities. Further, the high toxicity can cause a premature termination of the MTX administration, which decreases its potential efficiency [10]. In this study, we investigated the optimization of MTX dose adjustment as a potential factor to reduce MTX toxicity.

A three-compartment PK model of MTX is presented. Patient sex, age, BSA and SCr were related to CL. A 16% lower CL was estimated for females compared to males. Simulations using the final covariate model support dosing stratified for BSA quartiles.

The identification of clinically relevant covariates has been the main objective of numerous population PK evaluations of MTX, providing inconsistent findings on covariate relationships [24–34] . In contrast to our study, several previously published studies did not support a sex effect [24, 30–34]. Apart from differences in sample sizes, the particular combination of covariates in the model might have contributed to this inconsistency. For example, the inclusion of sex in a model containing SCr effect provided a distinct model improvement (OFV reduced by 32.0 points). In comparison, only a marginal improvement (OFV reduced by 4.40 points) resulted in the univariate evaluation (i.e., without considering any other covariates). This finding supports that a sex effect should be considered to account for differences in creatinine generation rates in male and female subjects. To quantify the contribution of sex effects beyond renal function, data on urinary excretion might be useful. However, such data was not available for patients in our database. The effect of sex on CL needs further investigation.

Age was related to MTX CL in a few studies [30, 32] while inconsistencies exist in the majority of studies [25, 34–38]. Some studies presented the influence of body weight and patient’s age on both the CL and V of MTX [31, 33]. Mei et al. showed that V of MTX increased with increasing age and supported the preference of age over body weight as a covariate influencing V. A relationship between weight and V was reported by some other studies as well [30, 31, 34, 39, 40]. Age was found to be significant on CL in our study with a ∆OFV of 13.0.

SCr was the most significant covariate with a ∆OFV of 191. This is in line with other studies, where MTX elimination was significantly correlated with SCr [34, 41, 42]. The observed effect is physiologically plausible as MTX is primarily eliminated by the kidney [24]. Nevertheless, the covariate relationship between SCr concentrations and MTX CL faces disagreements in other studies [30, 31, 33, 37].

The covariate analysis in the present evaluation was based on baseline covariate information due to missing covariate data during the treatment time course for a significant number of patients. The development of covariate models incorporating time-varying covariate data is a useful approach in general, as it may provide a better explanation of IIV and IOV of PK parameters and thereby improve the predictive ability of the model. This might be of particular interest if a population PK model is used for Bayesian TDM over a prolonged treatment course. However, we intended to use covariate data mainly to generate an initial idea on expected concentrations before administering the first dose. During the treatment course, concentration data becomes available, and covariate data is of less clinical relevance. If improper imputation methods are applied, a misspecified model and distorted predictions might result. Thus, we believe that a model comprising solely baseline covariate data provides advantages and might therefore be preferable over a model with time-varying covariate data.

Preference of BSA-based dosing over flat dosing or based on other measures, such as patient genotype / phenotype, is an ongoing debate. In contrast to the BSA-based dosing, flat dosing is proposed for several anticancer drugs where BSA has been shown not to reduce the random PK variability to a clinically relevant degree [43–45]. Apart from the simplified clinical handling of flat dosing, BSA-based dosing introduces additional uncertainties which are difficult to assess due to the arbitrary choice of BSA equation [46]. Therefore, BSA as a body size measure should ideally be avoided if precise dose calculations are intended. Furthermore, scaling doses with BSA is likely to provide implausibly low or high doses in subjects with exceptionally low or high BSA. Owing to the simplicity needed to implement body size-based dosing regimens in clinical practice, a direct, proportional relationship between BSA and dose is often assumed. This is contradictory to our current knowledge on physiology and PK and further adds to the uncertainties. Although MTX PK is linear, i.e. exhibits a proportional increase in exposure (in terms of AUC) with the increase in dose, it does not imply that MTX exposure is reciprocally proportional to BSA. In our study, the change of exposure attributable to BSA was smaller and we only observed relevant differences between BSA and flat dosing for patients with either very low or very high BSA. A stratified approach is a reasonable alternative to BSA-based dosing with individuals in the upper and lower BSA quartiles. A stratified approach is a reasonable alternative to BSA-based dosing with individuals in the upper and lower BSA quartiles. It is important to mention that the current findings are based on retrospective data and need to be further validated in a prospective study. It should be noted that these findings are conditional on the defined TDM target. The TDM target is a concentration threshold associated with overexposure, while no threshold for underexposure is currently available. To avoid possible underexposure, achieving 70–130% of the AUC for a subject with a typical BSA of 1.73m^2^ was used as an additional criterion in the simulation analysis. No pharmacokinetic/pharmacodynamic (PK/PD) target related to efficacy is part of the TDM at the University Hospital Cologne, and, to the best of our knowledge, no validated PK/PD target related to efficacy is currently available.

Apart from the covariate and BSA evaluation, a non-linear CL component was identified in this study. Non-linear elimination has been reported before and might be attributable to the transporter-mediated tubular secretion of MTX [47–49]. Despite the significant improvement of the model after inclusion of non-linear CL, the impact of the non-linear component on estimated exposure and the excess of TDM thresholds was negligible. Thus, non-linearity seems to be of minor clinical relevance in the current cohort of patients. This might change if additional targets, such as PK/PD targets related to efficacy, become available. In this case, the model with the non-linear component as presented in the Supplement might be re-evaluated. Furthermore, the non-linear CL component might have a more pronounced impact on PK in presence of genetic polymorphisms and when MTX is co-administered with substrates, inducers, or inhibitors of the relevant membrane transporters.

A major limitation of the current investigation is that the optimal target exposure regarding the efficacy of MTX in various malignancies is unknown. Data on minimum drug exposure needed to achieve a positive therapeutic outcome with minimal toxicity is currently scarce. Dedicated efforts are needed to draw conclusions based on the efficacy profile of the drug with respect to the underlying disease. Furthermore, TDM data is generally obtained from clinical practice and therefore provides a reduced data quality compared to clinical trial data. Although the data was checked carefully for inconsistencies, it cannot be precluded that errors in TDM procedures translate into model misspecifications.

## Conclusions

A three-compartment model described PK of MTX. A lower CL estimated for the female patients needs to be investigated in future studies. Plasma SCr, patient age, sex and BSA were found additionally as statistically significant covariates on CL. Stratified MTX dosing can be a reasonable alternative to BSA guided dosing.

## Supplementary Information

**Additional file 1:** **Supplementary Table.** Bootstrap population PK parameter estimates of the combined linear and nonlinear model obtained from bootstrap analysis. **Supplementary Figure 1.** Goodness of fit plots; A: observed vs individual predicted (IPRED) concentration (mg/L); B: observed vs population predicted (PRED) concentrations; C: conditional weighted residuals (CWRES) vs population predicted concentrations; D: conditional weighted residuals vs time after first dose (TAFD). Concentrations are presented on log scale in the upper panel. **Supplementary Figure 2.** Numerical predictive check comparing each observation with its own simulated distribution: Continuous line is the empirical cumulative distribution function of the observed concentrations. Dashed line with shaded area is the predicted cumulative distribution with 95% prediction interval computed from simulated data.

## Data Availability

The datasets used and/or analysed during the current study available from the corresponding author on reasonable request.
