# Supplementary material for: Evaluation of body-surface-area adjusted dosing of high-dose methotrexate by population pharmacokinetics in a large cohort of cancer patients
Source: BMC Cancer. 2021 Jun 20;21:719. doi: 10.1186/s12885-021-08443-x (PMC8214796; doi:10.1186/s12885-021-08443-x)

**Supplementary material**

**Supplementary Table:** Bootstrap population PK parameter estimates of the combined linear and nonlinear model obtained from bootstrap analysis

|  | **Mean** | **% RSE** | **95% CI** |
| --- | --- | --- | --- |
| **PK parameters** |  |  |  |
| LCL (L h^-1^) | 4.77 | 14.1 | 3.28 - 6.02 |
| Vmax (µmol h^-1^) | 2.46 | 31.6 | 0.96 - 4.19 |
| Km (µmol L^-1^) | 1.02 | 31.9 | 0.56 - 1.80 |
| V_1_ (L) | 1.12 | 32.5 | 0.42 - 1.67 |
| V_2_ (L) | 3.87 | 24.5 | 2.03 - 5.86 |
| V_3_ (L) | 5.08 | 30.2 | 2.12 - 7.70 |
| Q_1_ (L h^-1^) | 0.52 | 26.7 | 0.27 - 0.83 |
| Q_2_  (L/h) | 0.04 | 24.1 | 0.02 - 0.06 |
| **Covariate effects on CL** |  |  |  |
| SCr (mg^-1^ dL) | -0.91 | -12.0 | -1.11 - -0.68 |
| Age (year^-1^) | -0.23 | -37.0 | -0.39 - -0.05 |
| Sex (fractional decrease in females) | -0.28 | -21.9 | -0.39 - -0.14 |
| BSA (m^-2^) | - | - | - |
| **IIV (ω^2^)** |  |  |  |
| LCL | 0.07 | 28.9 | 0.034 - 0.11 |
| V_1_ | 2.127 | 58.8 | 0.46 - 4.43 |
| COV(LCL, V_1_) | 0.10 | 79.2 | 0.002 - 0.28 |
| **IOV (ω^2^)** |  |  |  |
| LCL | 0.07 | 28.9 | 0.034 - 0.11 |
| V_1_ | 2.13 | 58.8 | 0.46 - 4.43 |
| Vmax | 0.10 | 79.2 | 0.002 - 0.28 |
| **RUV (σ^2^)** |  |  |  |
| Additive error | 0.02 | 19.3 | 0.01 - 0.03 |
| Exponential error | 0.22 | 9.67 | 0.18 - 0.26 |

PK = pharmacokinetic, RSE = relative standard error, CI = confidence interval, CL = clearance, LCL = linear fraction of clearance, Vmax = maximum rate of elimination, Km = concentration at half-maximal rate, V_1_ = central volume of distribution, V_2_ and V_3_ = peripheral volumes of distribution, Q_1_ and Q_2_ = inter-compartmental clearances, AUC = Area under the curve, SCr = Serum creatinine, IIV = inter-individual variability, COV = covariance, IOV = inter-occasion variability, RUV = residual unexplained variability.

**Supplementary figure 1: Goodness of fit plots;** A: observed vs individual predicted (IPRED) concentration (mg/L); B: observed vs population predicted (PRED) concentrations; C: conditional weighted residuals (CWRES) vs population predicted concentrations; D: conditional weighted residuals vs time after first dose (TAFD). Concentrations are presented on log scale in the upper panel.


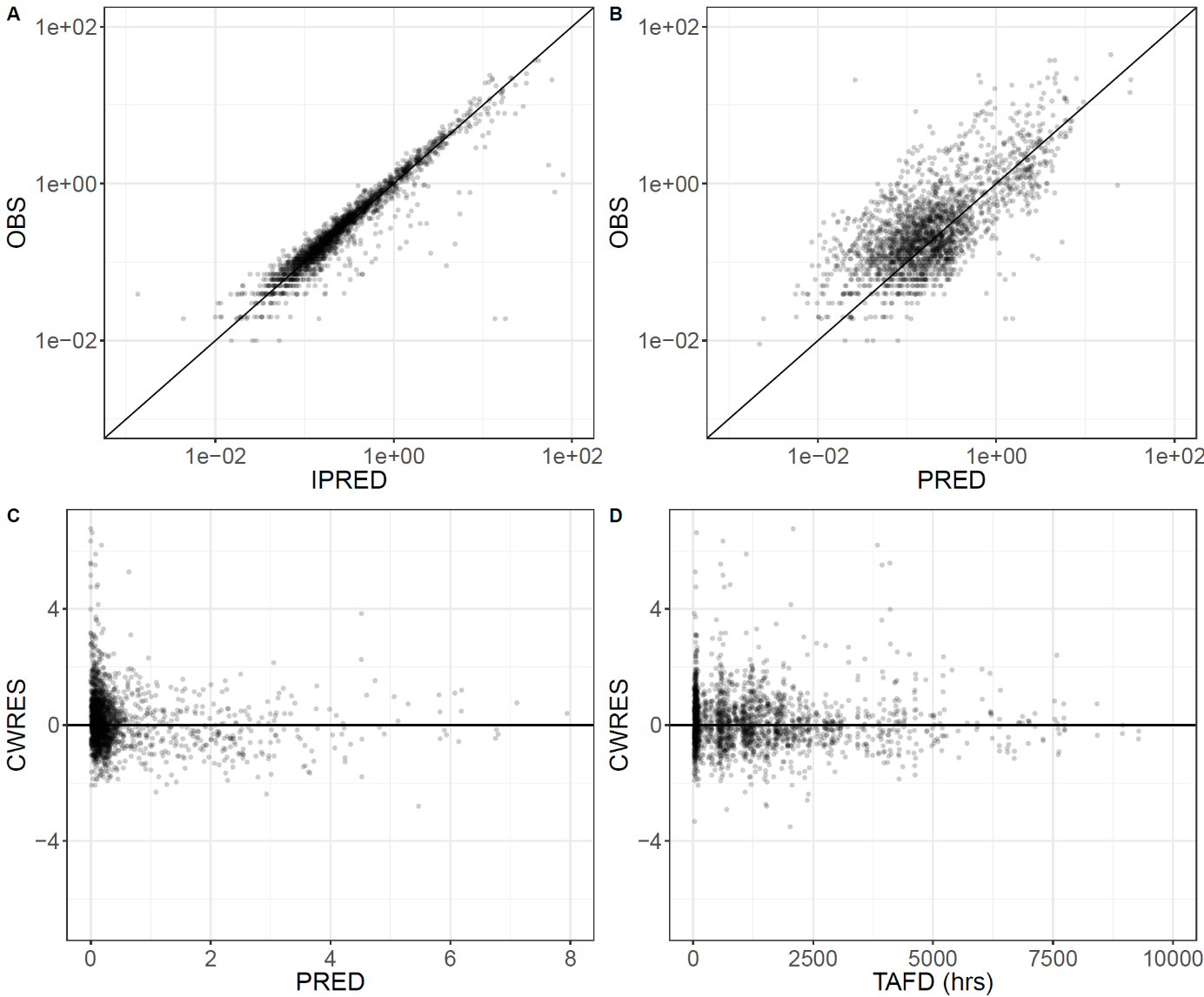


**Supplementary figure 2:** Numerical predictive check comparing each observation with its own simulated distribution: Continuous line is the empirical cumulative distribution function of the observed concentrations. Dashed line with shaded area is the predicted cumulative distribution with 95% prediction interval computed from simulated data.


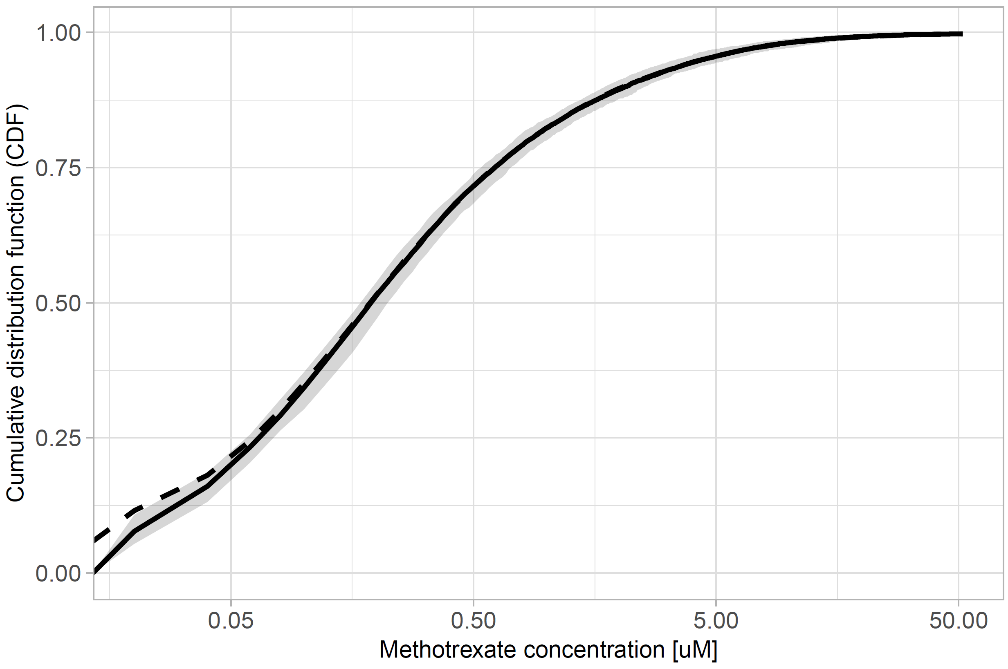

Supplement: Supplementary file 1 — Additional file 1: Supplementary Table. Bootstrap population PK parameter estimates of the combined linear and nonlinear model obtained from bootstrap analysis. Supplementary Figure 1. Goodness of fit plots; A: observed vs individual predicted (IPRED) concentration (mg/L); B: observed vs population predicted (PRED) concentrations; C: conditional weighted residuals (CWRES) vs population predicted concentrations; D: conditional weighted residuals vs time after first dose (TAFD). Concentrations are presented on log scale in the upper panel. Supplementary Figure 2. Numerical predictive check comparing each observation with its own simulated distribution: Continuous line is the empirical cumulative distribution function of the observed concentrations. Dashed line with shaded area is the predicted cumulative distribution with 95% prediction interval computed from simulated data. [file 12885_2021_8443_MOESM1_ESM.docx]
